# Attitudes of Mainstream and Special-Education Teachers toward Intellectual Disability in Italy: The Relevance of Being Teachers

**DOI:** 10.3390/ijerph17197325

**Published:** 2020-10-07

**Authors:** Laura Arcangeli, Alice Bacherini, Cristina Gaggioli, Moira Sannipoli, Giulia Balboni

**Affiliations:** Department of Philosophy, Social and Human Sciences, and Education, Università degli Studi di Perugia, Piazza G. Ermini, 1, 06123 Perugia, Italy; laura.arcangeli@unipg.it (L.A.); alice.bacherini@studenti.unipg.it (A.B.); cristina.gaggioli@gmail.com (C.G.); moira.sannipoli@unipg.it (M.S.)

**Keywords:** attitudes toward ID, intellectual disability, mainstream teachers, special-education teachers, ATTID, training, support

## Abstract

The attitudes of teachers toward intellectual disability (ID) contribute to an effective school inclusion of students with ID, thereby enhancing their quality of life. The present study was aimed at investigating the attitude differences toward ID of mainstream and special-education teachers in Italy and the general and specific teachers’ characteristics most related to these attitudes. An online version of the Attitudes toward Intellectual Disability (ATTID) questionnaire was filled by 307 mainstream teachers and 237 special-education teachers. The findings show that special-education teachers held more positive attitudes. Specific ATTID dimensions were positively affected for both types of teachers by previous training in special education/ID, perceived support, and promotion of positive attitudes toward ID, in addition to the quality of relationships with individuals with ID, while they were positively affected for special-education teachers by perceived efficacy of ID knowledge. No or very limited effects were observed for previous experience in teaching students with typical development or ID (even with severe/profound ID). Fostering resources to provide teachers with high-quality training, support, and resources and strategies to promote positive attitudes toward ID seems a relevant approach leading to favorable attitudes, thereby improving the quality of life of students with ID.

## 1. Introduction

Intellectual disability (ID) is a disorder characterized by significant limitations in both intellectual functioning and adaptive behavior, as expressed in conceptual, social, and practical skills, with onset before 18 years of age or during the developmental period [1,2,3]. Interventions for individuals with ID should be implemented to enhance their quality of life. Based on Schalock and Verdugo’s [4] model, quality of life is a multidimensional construct made up of eight core domains: personal development, self-determination, interpersonal relationships, social inclusion, rights, emotional well-being, physical well-being, and material well-being. Inclusive environments are environments that provide access to resources, information, and relationships and encourage growth and development, in addition to supporting people, accommodating psychological needs related to autonomy, competence, and relatedness [5]. These factors may improve all the dimensions of quality of life of individuals with ID.

Educational inclusion is an example of an inclusive environment. Educational inclusion implies not only the complete integration of children with ID or other special needs into programs and activities with peers [6], but also the planning of individualized projects for all students in order to promote the best opportunities for the personal growth of everyone [7,8,9]. Inclusion is an unending process of increasing learning, participation, and quality of life for all students, with or without ID or other special educational needs [10,11,12]. Social participation and school inclusion are rights for students with ID, as stated by several international documents, such as the World Declaration on Education for All [13], the Salamanca Statement [14], the No Child Left Behind American Law [15], or the Convention on the Rights of Persons with Disabilities [16]. They represent essential components for a life of quality and equality [17]. In Italy, since the end of the 1970s, Law 517 [18], Law 104 [19], and Law 96 [20] have established that students with ID, as all other students, have the right to attend mainstream education classes, supported by a special-education teacher for a number of hours per week [8,21].

An environmental factor that plays a central role in the effective social inclusion of students with ID is represented by the attitudes of teachers toward ID. Attitude is a personal evaluation toward people, objects, or events which generates either positive or negative judgments, consequently predisposing individual behavior [22]. The most used models of attitudes toward ID are based on the three-factor model of attitudes [23], which defines an attitude as a multidimensional construct represented by three dimensions: (a) cognitive (beliefs or knowledge of ID), (b) emotional (feelings created by ID), and (c) behavioral (predisposition to act toward individuals with ID).

Some studies found that teachers show more negative attitudes toward students with ID than toward those with other kinds of disabilities (e.g., physical or sensory), because students with ID need a greater level of support and adaptation for classroom activities [24,25,26]. Hence, studying the attitudes toward ID of teachers, as well as factors related to those attitudes, seems to be relevant; nevertheless, very few investigations were conducted on this topic [27,28,29,30]. In more detail, these studies investigated, in different countries and cultural contexts (i.e., Egypt, Turkey, Canada, and Scotland), the attitudes toward ID of elementary-school mainstream teachers or of middle-school physical education teachers. Moreover, factors related to teachers’ attitudes toward ID, particularly those concerning specific teacher characteristics, were investigated (e.g., self-efficacy, training in special education or ID, years of teaching experience). A higher level of teacher self-efficacy was found to be associated with more positive attitudes toward individuals with ID [30]. Furthermore, training in ID was found to be associated with more positive attitudes [28], especially concerning knowledge of the capacity and rights of individuals with ID, as well as the willingness to interact with persons with ID [29]. However, no relationship was found between having attended special-education classes and the attitudes toward ID of teachers [30]. Similarly, Sermier Dessemontet et al. [29], but not Ozer et al. [28], revealed an association between previous experience teaching pupils with ID and higher levels of willingness to interact with persons with ID, along with less discomfort. Moreover, Ozer et al. [28] found that teachers with fewer years of teaching experience showed more positive attitudes, while Wilson et al. [30] found the opposite result.

With respect to general characteristics contributing to teachers’ attitudes toward ID, it was found that prior contact with persons with ID [27], particularly, the quality rather than the quantity of prior contacts [29], is related to the attitudes of teachers. Indeed, Sermier Dessemontet et al. [29] found no associations between the frequency of contact and any dimension of the attitudes of teachers toward ID, while a higher quality of contact was found to be related to less discomfort toward individuals with ID and a higher willingness to interact with them. Finally, a younger chronological age of teachers was found to be associated with more positive attitudes toward ID [28].

More studies were realized on the attitudes of teachers toward school inclusion of students with ID or other disabilities. For example, it was found that teachers teaching at a lower school level and those with higher levels of perceived school support showed more positive attitudes toward the inclusion of pupils with ID or other disabilities [31,32,33]. However, other investigations did not find any association between school level and attitudes toward inclusion [34,35].

Given the Italian history with regard to the inclusion of individuals with ID and, therefore, the experience and contact of teachers with students with ID in Italy, it would be interesting to study teachers’ attitudes toward ID in Italy, as well as any factors related to those attitudes. To date, in Italy, studies were only realized highlighting teachers’ attitudes toward the inclusion of students with ID in mainstream classes [32,36]. It was found that special-education teachers have a better attitude than mainstream teachers, while mainstream teachers with experience in teaching pupils with ID had a better attitude and were not negatively affected by age and years of service. Moreover, mainstream high-school teachers and teachers older than 40 asked for more training [32].

The present study aimed to investigate the attitudes toward ID of mainstream teachers and special-education teachers in Italy, as well as the characteristics related to these attitudes. Firstly, we investigated the quality of the attitudes of mainstream and special-education teachers toward ID (positive, neutral, or negative) and if there were any differences between the attitudes of these two groups of teachers. Secondly, separately for mainstream teachers and special-education teachers, we investigated the relationships of attitudes toward ID with (a) the teacher’s personal characteristics (general characteristics, i.e., age, prior quantity, closeness, and quality of contact with individuals with ID) and (b) characteristics specific to being teachers (teacher-specific characteristics, i.e., school level taught, previous training in special education or ID, years of teaching experience, years of experience teaching pupils with ID, experience teaching pupils with severe/profound ID, perceived efficacy of ID knowledge, perceived support, and promotion of positive attitudes toward ID). Toward this aim, separately for mainstream and special-education teachers, we first investigated the relationship between each dimension of attitude toward ID (cognitive, emotional, and behavioral) and each individual’s general and teacher-specific characteristic; then, we investigated the degree to which an individual’s teacher-specific characteristics affected each dimension of attitude toward ID, in addition to the individual’s general characteristics. In this way, it was possible to identify the factors positively related to teachers’ attitudes toward ID, which need to be taken into account when planning interventions to create an inclusive environment for students with ID.

## 2. Materials and Methods

This study was conducted following the ethical standards laid down in the 2013 Fortaleza version of the Declaration of Helsinki, it was a voluntary survey for adults that were not in a vulnerable condition, written informed consent was obtained from each participant, and their anonymity was guaranteed. The participants did not receive any form of incentive to participate in this study. Given that, being not a clinical study and being the participants not in a vulnerable condition, the present study is not subject to the prior ethical approval of the Ethical Committee in accordance with the Regulation of the European Parliament n. 536/2014. 

### 2.1. Instruments

#### 2.1.1. Attitudes toward Intellectual Disability

The Attitudes Toward Intellectual Disability Questionnaire (ATTID) [37] was used to measure attitudes toward ID. It is composed of 67 items structured in 5 dimensions that map the three-factor model of attitudes [22,23]: (a) two dimensions related to a cognitive factor measured by the knowledge of capacity and rights (20 items) and the knowledge of causes of ID (seven items); (b) two dimensions related to an affective factor measured by discomfort (17 items) and sensitivity/tenderness (6 items); (c) a dimension related to a behavioral factor measured by interaction (17 items). The discomfort dimension assesses the feelings of stress, fear, embarrassment, anxiety, or inadequacy toward persons with ID. The knowledge of capacity and rights dimension measures myths and beliefs related to ID and the rights of individuals with ID, such as the right to attend school, to work, to have a romantic partner, to be integrated into the community. It also concerns these individuals’ ability and potential. The interaction dimension investigates willingness to interact with people with ID. The sensitivity/tenderness dimension evaluates the presence of feelings of pity, sadness or compassion toward persons with ID. Finally, the knowledge of causes dimension measures the knowledge of the etiology of ID. Respondents were asked to express their level of agreement/disagreement with each statement, according to a 5-point Likert scale ranging from 1 = totally agree to 5 = totally disagree. Higher scores indicate more negative attitudes toward individuals with ID.

We developed an Italian translation and adaptation of the ATTID (available upon request) in agreement with the International Test Commission Guidelines for Translating and Adapting Tests [38]. In the interaction dimension, one item was split into two items, and two more items were added in order to better evaluate the Italian school-level context (see Supplementary Materials, Note S1). As a result, the Italian ATTID consists of 70 items.

Psychometric properties of the Italian ATTID were investigated using the data collected in the present study. The factorial structure was preliminarily studied via Confirmatory Factor Analyses (CFA), using data from 485 of the 544 teachers of the present investigation (59 participants were excluded because they presented multivariate outliers for this analysis). The robust maximum likelihood estimator (MLR) was used, given that the items’ score distributions were far from being multivariate normal (based on Mardia’s test) [39]. A first CFA was carried out using the normalized score obtained for the 70 items of the ATTID. The latent variables were the five ATTID dimensions, and the observed variables were the 70 items; each of them loaded only the corresponding latent variable. However, two of the added items of the interaction dimension—those regarding the teachers’ opinion about whether individuals with ID should attend regular preschool or middle school—had the same answers as the original items regarding attending primary school. Therefore, the two added items were deleted. Moreover, two items of the sensitivity/tenderness dimension—those regarding if individuals felt empathy or sympathy for an individual with ID that they met on the street—had no correlation with the other items of the dimension and did not load the corresponding dimension. Therefore, these two items were eliminated. A second CFA was run with the 66 remaining items loading the corresponding dimension. We found that all factor loadings of each item on the corresponding dimension were statistically significant and ranged from 0.323 to 0.879 (mean = 0.605; median = 0.602). The ATTID dimensions were correlated, with inter-correlation coefficients ranging from 0.18 to 0.69 for the discomfort, knowledge of capacity and rights, interaction, and sensitivity/tenderness dimensions; the inter-correlation coefficients ranged from -0.08 to 0.18 for the knowledge of causes of ID and the other four dimensions. The goodness-of-fit indexes were acceptable concerning the root-mean-square error of approximation (RMSEA = 0.069) and the standardized root-mean-square residual (SRMR = 0.092), but poor concerning the comparative fit index (CFI = 0.749), and the Tucker–Lewis index (TLI = 0.737) [39,40]. Data collection is in progress to increase the number of participants and make the sample less homogeneous.

Internal consistency was found to be adequate to excellent [41], with Cronbach’s alpha coefficients of 0.93 for the discomfort dimension, of 0.91 for both the knowledge of rights and the interaction dimensions, of 0.86 and 0.75 for the sensitivity/tenderness and the knowledge of causes dimensions, respectively, and of 0.94 for the total scale. The construct validity was also investigated by computing the inter-correlation coefficients for the five ATTID dimensions, which produced results very similar to those reported for the original version [37]. The magnitude of the correlation coefficients was evaluated as trivial (<0.10), small (0.10–0.29), medium (0.30–0.49), large (0.50–0.69), or very large (≥0.70) [42]. The correlation coefficients for discomfort, knowledge of capacity and rights, interaction, and sensitivity/tenderness dimensions were small to large, ranging from Pearson’s *r* = 0.17 to *r* = 0.69. The correlations coefficients of knowledge of causes of ID with the other four ATTID dimensions were trivial or small, ranging from Pearson’s *r* = −0.09 to *r* = 0.12.

#### 2.1.2. Individuals’ General Characteristics

Questions were adapted from the ATTID or developed for investigating the following individuals’ general characteristics that are generally investigated in studies regarding attitudes toward ID (see Supplementary Materials, Table S1): age (range: 1–5); quantity of contact with individuals with ID, split into (a) the number of known individuals with ID and (b) the frequency of contact with individuals with ID, which were collapsed into a unique variable called the quantity of contact with individuals with ID (range: 1–7; Cronbach’s alpha = 0.63); closeness of relationships with individuals with ID (range: 1–4); quality of relationships with individuals with ID (range: 1–3). Higher scores indicated higher levels of the measured characteristic.

#### 2.1.3. Individuals’ Teacher-Specific Characteristics

Questions were developed for investigating the following individuals’ teacher-specific characteristics (see Supplementary Materials, Table S2): school level taught (range: 1–4); past training in special education or ID, split into (a) type of specialization for special-education activities, based on which we classified the years of training in special education, and (b) hours of courses attended on ID, which we then collapsed into a unique variable called training in special education or ID (range: 1–7; Cronbach’s alpha = 0.52); years of teaching experience (range: 1–4); years of experience teaching pupils with ID (range: 1–4); experience teaching pupils with severe or profound ID (score: 0, 1). Higher scores represented higher levels of the measured characteristic.

Moreover, we developed an additional pool of 20 items (see Supplementary Materials, Table S2) to thoroughly investigate aspects related to teachers’ perceived efficacy of their own knowledge of ID or in handling pupils with ID, teachers’ perceived support received from the school and community context for teaching pupils with ID, and efficacy of strategies applied in the classroom to promote positive attitudes toward ID and inclusion of students with ID by the school. The rating scale of the items was the same 5-point Likert scale as for the ATTID; however, for the analyses of the present study, the scoring system was reversed, such that higher scores represented higher levels of the measured constructs.

A principal component analysis (PCA) with a Promax rotation was carried out using the normalized score obtained for these 20 items, using data from 515 of the 544 teachers of the present investigation (29 participants were excluded because they presented multivariate outliers for this analysis) [43]. The Kaiser–Meyer–Olkin (KMO) test of sampling adequacy was meritorious, corresponding to a score of 0.87 [44], while Bartlett’s test of sphericity indicated that the variables were sufficiently related to one other (*χ^2^* = 5079.61; *p* < 0.001). The number of components to be extracted was identified considering (a) the Kaiser–Guttman criterion, (b) the scree test, and (c) the results of a parallel analysis. Both the Kaiser–Guttman criterion and the parallel analysis suggested five components to be extracted. However, the inspection of the scree plot showed a curve inflection point after the third component, thereby justifying a three-component solution. A PCA with five components showed only two items loading on the fifth component, while a PCA with four components presented an item overlapping two different components. On the contrary, the three-component solution seemed most adequate (accounting for 53.67% of the variance), with at least three items loading on each of the three components, along with no bifactor items and item factor loadings ranging from 0.42 to 0.92. The components were labeled as (a) perceived efficacy of ID knowledge (eight items), (b) perceived support (seven items), and (c) promotion of positive attitudes toward ID (five items). Internal consistency was investigated by computing Cronbach’s alpha, resulting in coefficients of 0.94, 0.80, and 0.53, respectively. Given the small number of items for the third component, its Cronbach’s alpha coefficient may be considered acceptable [45].

#### 2.1.4. Social Desirability

The Balanced Inventory of Desirable Responding, Short Form (BIDR-6) [46,47] was used to detect attempts at simulation. This measure comprises 16 items with a 6-point Likert scale, ranging from 1 = strongly disagree to 6 = strongly agree, which evaluate the unconscious tendency to provide honest but positively biased responses, as well as the habitual and conscious presentation of a favorable public image. Individuals with a total score exceeding the 95th centile of the normative sample were identified as simulators. An Italian adaptation was used with adequate reliability and validity [46].

### 2.2. Participants

Participants were 544 Italian teachers (87% females, 11% males, and 2% who did not answer), of which 307 were mainstream teachers (90% females, 9% males, and 1% who did not answer) and 237 were special-education teachers (83% females, 14% males, and 3% who did not answer). Overall, they were recruited from 19 out of 20 Italian regions, representing 73 out of 107 Italian provinces. The schools in which they worked were located in cities with the following numbers of inhabitants (in thousands): <5 (16%), 5–20 (29%), 20–50 (19%), 50–100 (10%), 100–250 (15%), 250–500 (3%), or >500 (8%). The majority of teachers reported that their school had regular contact with local medical services (91%) and that psychologists, pedagogists, or other professionals, such as social workers or speech therapists, were available at their school (77%). Sixty-one percent of the teachers had previously attended classes on issues related to ID (e.g., inclusive education).

Originally, 691 participants were recruited; they were mainstream teachers and special-education teachers, as well as other professionals who worked in the school (e.g., school heads, administrative or technical clerks, janitors, social workers, and aides). However, 43 individuals were excluded because they did not complete the entire questionnaire (*n* = 14), because they were retired teachers (*n* = 8), trainee teachers (*n* = 13), or teachers in a foreign country (*n* = 3), or because they were not working consistently at school (*n* = 5). Then, of the remaining 648 individuals, 49 were excluded as potential simulators because they exceeded the cut-off score for social desirability, as measured by the BIDR-6. As a consequence, 599 participants remained: 307 mainstream teachers, 237 special-education teachers, and 55 other school professionals. Due to its small size, we decided to exclude the latter group, focusing our study only on the 307 mainstream and 237 special-education teachers; their characteristics are reported in Table 1.

Concerning the study population, based on a description of Italian classroom teachers and academic staff investigated in 2018 by the Eurostat [48], i.e., the statistical office of the European Union, the study population was mostly female (81%), relatively mature (2% aged 18–29; 11% aged 30–39; 28% aged 40–49; 40% aged 50–59; and 19% aged 60 or more), teaching at preschool (12%), elementary school (31%), middle school (22%), or high school (35%). The participants of this study were not selected randomly but based on voluntary responses. However, there was no difference between them and the study population in terms of gender (χ^2^_(1)_ = 2.06, *p* = 0.15), age (χ^2^_(4)_ = 0.01, *p* = 0.99), and school level taught (χ^2^_(3)_ = 0.92, *p* = 0.82).

### 2.3. Procedure

An online questionnaire, including the items of the ATTID Italian version, the questions we adapted or developed for measuring the individuals’ general and teacher-specific characteristics, and the items of the BIDR-6, was arranged with Google Forms. Data collection took place between May 2018 and April 2019. The online questionnaire was disseminated via the most popular Facebook groups and an online magazine of mainstream and special-education teachers (i.e., Orizzonte Scuola). Moreover, it was spread by teachers who attended the one-year post-lauream master’s program in special education provided by the University of Perugia in 2018 and by university professors affiliated with the Italian Association of Special Education (SIPeS).

### 2.4. Data Analysis

As a prerequisite for all analyses, the score distribution in each ATTID dimension and the metric variables that measured individual characteristics (see Table 1) were checked for mainstream teachers and special-education teachers, considering the following factors: (a) the presence of univariate outliers, i.e., participants with a *z*-value higher than |3.29|, and multivariate outliers, i.e., participants for which the probability associated with the Mahalanobis distance was lower than 0.001; (b) univariate normality, i.e., skewness and kurtosis between −1.00 and 1.00, and multivariate normality, i.e., Mardia’s test being negative.

To investigate the quality of the attitudes of mainstream and special-education teachers toward ID, descriptive statistics were computed for each of the five ATTID dimensions, with scores expressed on a 5-point Likert scale (i.e., raw score relative to the number of the corresponding items). Given that higher scores indicated more negative attitudes toward individuals with ID, as suggested by the ATTID’s authors [37], scores of 1 (totally agree) and 2 (agree) were evaluated as positive attitudes, a score of 3 (neither agree nor disagree) indicated neutral attitudes, and scores of 4 (disagree) and 5 (totally disagree) represented negative attitudes.

To study if there were any differences in the five ATTID dimensions between mainstream and special-education teachers, a one-way multivariate analysis of covariance (MANCOVA) was performed. The type of teacher, i.e., mainstream or special-education teacher, was taken as the independent variable, while the five ATTID dimensions were introduced simultaneously as dependent variables. Years of teaching experience was introduced as a covariate variable to properly take into account differences in this variable between mainstream and special-education teachers (see Section 3). In the case of statistically significant differences, Cohen’s *d* effect size was computed and evaluated as negligible (<0.19), small (0.20–0.49), medium (0.50–0.79), or large (≥0.80) [49].

Before running the MANCOVA analysis, we checked for the following: (a) adequacy of the number of participants, verifying that the ratio between the size of the two groups did not exceed 10:1; (b) homogeneity of variance–covariance matrices trough Box’s *M* test; (c) homogeneity of the variance of each dependent variable through the Levene’s test; (d) linearity of the relationships of each dependent variable with the covariate variable; (e) homogeneity of the regression slopes [41]. Moreover, we investigated whether the two groups of teachers were paired for gender, performing a *χ^2^* test, as well as for age and years of teaching experience, performing Mann–Whitney tests. In the case of statistically significant differences, phi and rank-biserial correlation *rg* effect sizes were computed for *χ^2^* and Mann–Whitney tests, respectively; they were both evaluated as negligible (<0.10), small (0.10–0.29), medium (0.30–0.49), or large (≥0.50) [49,50].

To study the relationship between each individual’s general and teacher-specific characteristics and each ATTID dimension, correlation coefficients were computed for each group of teachers. The type of correlation coefficient depended on the measurement level of the individual’s characteristics. We computed Pearson’s correlation coefficient for the metric variables (i.e., age, quantity of contact with individuals with ID, training in special education or ID, perceived efficacy of ID knowledge, perceived support, and promotion of positive attitudes toward ID), Spearman’s correlation coefficients for the ordinal variables (i.e., closeness and quality of relationships with individuals with ID, school level taught, years of teaching experience, and years of experience teaching pupils with ID), and point-biserial correlation coefficients for the dichotomous variable (experience teaching pupils with severe/profound ID). The magnitude of the correlation coefficients was evaluated as trivial (<0.10), small (0.10–0.29), medium (0.30–0.49), large (0.50–0.69), or very large (≥0.70) [42].

Finally, to study the degree to which the individual’s teacher-specific characteristics affected each ATTID dimension, in addition to the individual’s general characteristics, we ran one hierarchical multiple regression for each of the five ATTID dimensions within each group of teachers. All general characteristics (i.e., age, quantity of contact, and closeness and quality of relationships with individuals with ID) and teacher-specific characteristics (i.e., school level taught, training in special education or ID, years of teaching experience, years of experience teaching pupils with ID, experience teaching pupils with severe/profound ID, perceived efficacy of ID knowledge, perceived support, and promotion of positive attitudes toward ID) that were found to have a statistically or tendentially significant correlation (*p* ≤ 0.08) with the considered ATTID dimension were entered as independent variables. At step 1, we introduced only an individual’s general characteristics, while, at step 2, we introduced also the individual’s teacher-specific characteristics. The *sr^2^* incremental, i.e., a modification of *R^2^*, was examined to detect the contribution of an individual’s teacher-specific characteristics affecting each ATTID dimension, in adjunct with the individual’s general characteristics. A simple linear regression was performed for mainstream teachers between the promotion of positive attitudes toward ID as an independent variable and the knowledge of causes dimension as a dependent variable, because only this factor was significantly related to this ATTID dimension. As an effect size for each significant independent variable, *f^2^* was computed and evaluated as small (0.02–0.14), medium (0.15–0.34), or large (≥0.35) [49]. Given the high number of comparisons with the same participants, Benjamini and Hochberg’s correction for multiple comparisons [51] was applied; however, the appropriate level of significance remained at *p* < 0.05.

Before running the regression analysis, assumptions were ascertained [43]. For mainstream and special-education teachers, we verified (a) the appropriateness of the number of participants, in accordance with the assumption that *n* ≥ 104 + *m* (where *m* is the number of independent variables); (b) the absence of multicollinearity among independent variables by computing the tolerance index, which should be higher than 0.05, and the variance inflation factor (VIF), which should be lower than 2; (c) the normality, linearity, and homoscedasticity of errors, by examining the shape of the residual distribution scatterplots; (d) the independence of errors, through the Durbin–Watson statistics; and (e) the absence of outliers in standardized residuals.

Only participants who reported never having had any relationship with individuals with ID were excluded from the correlation and regression analyses involving the variables related to closeness and quality of relationships with individuals with ID.

Statistical power of the hierarchical multiple regression analyses run was conducted with G*Power 3.1 software (Heinrich Heine Universität, Düsseldorf, Germany) [52]. With more detail, we ran post-hoc power analyses [49] for linear multiple regression fixed model with *R^2^* increase [53].

## 3. Results

For both mainstream and special-education teachers, neither univariate nor multivariate outliers were found, and the normality of the univariate and multivariate distributions of the scores for each ATTID dimension and for each metric characteristic were generally satisfied. Only the variables perceived efficacy of ID knowledge for both mainstream and special-education teachers and training in special education or ID and promotion of positive attitudes toward ID for special-education teachers presented a slight kurtosis. Therefore, correlation and regression analyses were performed with normalized scores of each metric variable.

### 3.1. Attitudes toward ID of Mainstream Teachers and Special-Education Teachers

#### 3.1.1. Quality of Attitudes toward ID

Table 2 presents the means (SD) of the raw scores and the 5-point Likert scale scores (i.e., raw score relative to the number of the corresponding items) obtained for mainstream and special-education teachers for each ATTID dimension. A higher score for an ATTID dimension indicates a more negative attitude. Considering the Likert scale, given that 1 and 2 represented positive attitudes, 3 represented neutral attitudes, and 4 and 5 represented negative attitudes, both mainstream teachers and special-education teachers had positive attitudes for all ATTID dimensions.

#### 3.1.2. Differences in Attitudes toward ID between Mainstream Teachers and Special-Education Teachers

The results of non-parametric statistics showed that special-education teachers, compared with mainstream teachers, were more often male (*χ^2^_(1)_* = 3.94, *p* < 0.05; *phi* = 0.09) and younger (*U* = 25,724, *z* = −6.14, *p* < 0.001; *rg* = 0.29), with fewer years of teaching experience (*U* = 21,510, *z* = −8.519, *p* < 0.001; *rg* = 0.41).

MANCOVA assumptions were satisfied, except for the homogeneity of variance–covariance matrices, given that Box’s *M* test showed statistically significant results (*F*_(15,1031317)_ = 1.68, *p* < 0.05). However, Box’s *M* test is so sensitive that some authors proposed setting the level of significance at *p* < 0.005 [54]. Levene’s test for homoscedasticity showed a significant result only for the dependent variable discomfort (*F*_(1,542)_ = 7.11, *p* < 0.01). Given the results of the Box’s *M* and Levene’s tests, we used Pillai’s trace criterion to report the multivariate test results, which is robust to these violations [55].

MANCOVA showed a multivariate statistically significant effect of the type of teacher on the attitude toward ID, controlled for years of teaching experience (Pillai’s trace = 0.023, *F*_(5,537)_ = 2.48, *p* < 0.05). The subsequent univariate tests showed that special-education teachers, compared with mainstream teachers, obtained statistically significant lower scores, i.e., more positive attitudes, for the ATTID dimensions discomfort (*F*_(1,541)_ = 10.80, *p* < 0.01), interaction (*F*_(1,541)_ = 7.13, *p* < 0.01), and sensitivity/tenderness (*F*_(1,541)_ = 5.56, *p* < 0.05). The effect size was small in all cases, with Cohen’s *d* equal to 0.29, 0.25, and 0.21, respectively. No statistically significant differences were found for the ATTID dimensions knowledge of capacity and rights and knowledge of causes of ID.

### 3.2. Relationships between General and Teacher-Specific Characteristics and Each Attitudes toward ID Dimension for Mainstream Teachers and Special-Education Teachers

#### 3.2.1. Correlation between Characteristics and Attitudes toward ID Dimensions

Table 3 reports the correlation coefficients between the scores for variables measuring an individual’s general and teacher-specific characteristics and each ATTID dimension. As can be seen, regarding an individual’s general characteristics, younger age was significantly related to a greater knowledge of capacity and rights for both types of teachers and to more interactions for special-education teachers only. A higher quantity of contact and a better quality of relationship with individuals with ID were significantly correlated with less discomfort and sensitivity/tenderness and more interactions for both types of teachers and with a greater knowledge of causes of ID for special-education teachers only. A closer relationship with individuals with ID was associated with more interactions for mainstream teachers and with a greater knowledge of causes of ID for special-education teacher.

Regarding an individual’s teacher-specific characteristics, teaching at a higher school level was related to a greater knowledge of capacity and rights for mainstream teachers only. More training in special education or ID was associated with less discomfort and sensitivity/tenderness for both types of teachers and with more interactions for mainstream teachers only. More years of teaching experience was related to a lesser knowledge of capacity and rights for both types of teachers and to few interactions for mainstream teachers only. More years of experience teaching pupils with ID and of experience teaching pupils with severe/profound ID was related to a lesser knowledge of capacity and rights for special-education teachers and to less sensitivity/tenderness for mainstream teachers. For mainstream teachers only, experience teaching pupils with severe/profound ID was related to less discomfort.

Higher levels of perceived efficacy of ID knowledge, perceived support, and promotion of positive attitudes were associated with more interactions for both types of teachers and with less discomfort for special-education teachers only. A higher perceived efficacy of ID knowledge was related to less sensitivity/tenderness for both types of teachers and to less discomfort for mainstream teachers only. Higher perceived support was related to a greater knowledge of capacity and rights for special-education teachers only. Finally, a higher promotion of positive attitudes was related to a greater knowledge of capacity and rights and of causes of ID for both types of teachers.

All these significant correlation coefficients had a small magnitude, except for some obtained for the quantity of contact and the quality of relationships with individuals with ID, as well as for the perceived efficacy of ID knowledge, which had a medium magnitude.

#### 3.2.2. Regressions of Characteristics on Attitudes toward ID Dimensions

To study the degree to which an individual’s teacher-specific characteristics affected each ATTID dimension, in addition to the individual’s general characteristics, we ran one hierarchical multiple regression for each of the five ATTID dimensions within each group of teachers. All the individual’s general and teacher-specific characteristics that were found to have a statistically or tendentially significant correlation (*p* ≤ 0.08) with the considered ATTID dimension were entered as independent variables. Each ATTID dimension was taken as the dependent variable.

With regard to the regression assumptions, particularly, the appropriateness of the number of participants, a maximum of eight and seven characteristics was entered as independent variables for mainstream teachers and special-education teachers, respectively. The minimum required numbers of 112 (i.e., 104 + number of independent variables = 104 + 8 = 112) and 111 (i.e., 104 + number of independent variables = 104 + 7 = 111) participants for mainstream and special-education teachers, respectively, were lower than the actual numbers of participants in each group (equal to 307 and 237, respectively); therefore, the assumption of adequacy of the number of participants was satisfied. Moreover, within each teacher group, the absence of multicollinearity among the independent variables was ascertained, because the tolerance and the VIF index values were higher than 0.50 and lower than 2, respectively. For each independent variable taken separately, as well as within the set of predictors, the normality, linearity, and homoscedasticity of the residuals were ascertained. Indeed, an examination of the shape of the residual distribution scatterplots revealed that, in all cases, the residuals were normally distributed around each and every dependent variables’ predicted score; residuals had a horizontal line relationship with the predicted dependent variables’ scores—hence, the shape of the scatterplots appeared rectangular—and the variance of residual scores was approximately equal for all predicted dependent variables’ scores [43]. The independence of errors in the regression solutions was satisfied too, because the Durbin-Watson values ranged from 1.75 to 2.13 and therefore fell within the suggested range of 1.5–2.2 [43]. Finally, no outliers in the standardized residuals were detected, given that no standardized residuals exceeded SD = 3.29 [43].

Table 4 represents the results of the regression analyses, and ***β*** (i.e., standardized beta regression coefficients), adjusted *R^2^* (i.e., explained variance), *F* (i.e. ANOVA F value of the overall significance of the regression), and *sr^2^* incremental are reported. Step 1 shows which of the individual’s general characteristics affected each ATTID dimension for mainstream and special-education teachers. Step 2 shows which of these individual’s general characteristics persisted in affecting the ATTID dimensions when considered simultaneously with the individual’s teacher-specific characteristics, which of the individual’s teacher-specific characteristics affected each ATTID dimension, and the degree to which the individual’s teacher-specific characteristics affected each ATTID dimension, in addition to the individual’s general characteristics (i.e., *sr^2^* incremental).

Step 2 shows that an individual’s teacher-specific characteristics improved the precision of score prediction for the majority of ATTID dimensions for both types of teachers. Specifically, for special-education teachers only, a higher perceived efficacy of ID knowledge positively affected the discomfort (*f^2^* = 0.12, power = 0.99) and interaction (*f^2^* = 0.06, power = 0.86) dimensions. For both mainstream teachers and special-education teachers, higher perceived support positively affected the interaction dimension (*f^2^* = 0.05, power = 0.87, for mainstream teachers; *f^2^* = 0.06, power = 0.86, for special-education teachers), higher promotion of positive attitudes positively affected the knowledge of causes of ID dimension (*f^2^* = 0.06, power = 0.99, for mainstream teachers; *f^2^* = 0.04, power = 0.73, for special-education teachers), and higher training in special education or ID positively affected the sensitivity/tenderness dimension (*f^2^* = 0.04, power = 0.81, for mainstream teachers; *f^2^* = 0.06, power = 0.83, for special-education teacher). Lastly, for mainstream teachers only, a higher school level taught positively affected the knowledge of capacity and rights dimension (*f^2^* = 0.02, power = 0.43), and more years of teaching experiences negatively affected the interaction dimension (*f^2^* = 0.02, power = 0.43).

Finally, regarding the individual’s general characteristics affecting attitudes when considered simultaneously with an individual’s teacher-specific characteristics, a higher age was found to negatively affect the knowledge of capacity and rights dimension (*f^2^* = 0.07, power = 0.96) for mainstream teachers and the interaction dimension (*f^2^* = 0.04, power = 0.68) for special-education teachers. A higher quantity of contact positively affected the discomfort (*f^2^* = 0.13, power = 1.00) and sensitivity/tenderness (*f^2^* = 0.04, power = 0.81) dimensions for mainstream teachers only. Closer relationships with individuals with ID positively affected the knowledge of causes of ID dimension (*f^2^* = 0.02, power = 0.41) for special-education teachers. Finally, for both types of teachers, a better quality of relationships with individuals with ID positively affected the discomfort dimension (*f^2^* = 0.22, power = 1.00, for mainstream teachers; *f^2^* = 0.05, power = 0.79, for special-education teachers) and the interaction dimension (*f^2^* = 0.12, power = 1.00, for mainstream teachers; *f^2^* = 0.13, power = 1.00, for special-education teachers), while it positively affected the sensitivity/tenderness dimension (*f^2^* = 0.09, power = 0.99) for mainstream teachers only.

The values of *f^2^* showed a small effect size for all predictors in both groups of teachers, except for the contribution of the quality of relationships with individuals with ID to the discomfort dimension for mainstream teachers, for which the effect size was medium. The explained variance by the factors inserted at Step 2 ranged from 27% (discomfort dimension of mainstream teachers) to 5% (sensitivity/tenderness and knowledge of causes of ID dimensions of special-education and mainstream teachers, respectively), confirming that the explanation power of the independent variables over the dependent variable was very limited.

## 4. Discussion

The present study was aimed at investigating the attitudes toward ID of Italian mainstream teachers and special-education teachers and verifying if there were any differences between them. In agreement with a previous study [29], both mainstream and special-education teachers reported positive attitudes; however, special-education teachers were found to be more willing to interact with individuals with ID, feeling less pity and discomfort. This outcome may be due to the higher preparation in teaching students with ID and the closer relationships with individuals with ID exhibited by special-education teachers compared to mainstream teachers [26,32]. The two teacher groups were matched for years of teaching experience but not for chronological age, leading to statistically significant differences, albeit with a small effect size. Therefore, the better attitude of the special-education teachers could also be due to their lower age, given that studies found a better attitude of younger teachers [28]. The two teacher groups were also different for gender, but previous studies did not reveal any effect of gender on teachers’ attitudes toward ID [28].

We also found that an individual’s teacher-specific characteristics overall affected the attitude toward ID, in addition to the individual’s general characteristics. Specifically, for both mainstream teachers and special-education teachers, we found that teachers with a higher level of training in special education or ID showed less pity, sadness, or compassion toward persons with ID, in agreement with previous investigations [28,29], while teachers with higher levels of promotion of positive attitudes toward ID had a better knowledge of causes of ID, in line with a previous study [30]. Furthermore, teachers with higher perceived support from the school and community contexts had greater willingness to interact with persons with ID, in line with a previous study about the inclusion of students with disabilities [31]. The magnitude of the effect sizes was similar for mainstream and special-education teachers, indicating that these characteristics were equally important for both types of teachers. Only special-education teachers with higher levels of perceived efficacy of ID knowledge showed less discomfort and were more willing to interact with individuals with ID. These findings might indicate that they feel confident in their competence and skills with regard to managing students with ID, and this security has positive effects on their attitudes toward ID [29,30]. Only mainstream teachers teaching at higher school levels showed a greater knowledge of capacity and rights of individuals with ID. Teachers at a higher school level, compared to those at a lower school level, might have had experience with students with a defined support system, who would, thus, be included in the classroom and in the community and be able to express their needs and show their strengths.

On the contrary, in spite of the relationships found with the correlation analyses among years of teaching experience, years teaching students with ID, and experience teaching pupils with severe/profound ID and the specific dimensions of attitudes, the regression analyses showed that, when considered simultaneously with all other characteristics, these factors were not associated with any attitude dimension for both type of teachers. The only exception was for mainstream teachers with fewer years of teaching experience, who showed more willingness to interact with individuals with ID. Our findings are in agreement with Ozer et al. [28], who found the same kind of relationships between attitudes and teaching experience but not with experience teaching pupils with ID, while they disagree with Sermier Dessemontet et al. [29], who found an association between attitudes of teachers toward ID and experience teaching students with ID.

Among all the individual’s general characteristics that affected attitude toward ID, when taken into account simultaneously with teacher-specific characteristics, the quality of relationships with individuals with ID affected a greater number of dimensions, along with having a higher effect size. Indeed, both types of teachers with a better quality of contacts felt less discomfort toward persons with ID and less reluctance when interacting with them, and only mainstream teachers had fewer feelings of sadness and pity. The magnitude of effect sizes was similar for both types of teachers, except for the higher effect size found with regard to discomfort for mainstream teachers, indicating that, particularly for this type of teacher, the quality of the relationships with students with ID is relevant to better interact with them without discomfort. On the contrary, the quantity of contact with individuals with ID positively affected discomfort and sensitivity/tenderness for mainstream teachers only, supporting previous findings indicating that the quality of contact with individuals with ID is more relevant than the frequency of contact [29].

Only special-education teachers with a closer relationship with persons with ID had a better knowledge of causes of ID. This result may be explained by the previous training in ID of special-education teachers, which allows them to know better the literature on ID and the possible causes of this disorder.

Finally, in agreement with a previous study [28], we found that younger mainstream teachers and special-education teachers had a better knowledge of the capacity and rights of persons with ID or were more willing to interact with them, respectively. These findings might be due to the better quality of training in special education or ID for younger mainstream teachers and to the greater motivation and willingness of younger special-education teachers.

The uniqueness of the present study consists of having studied the individual characteristics related to attitudes toward ID of both mainstream and special-education teachers, while simultaneously considering general characteristics, such as previous contact with individuals with ID and teacher’s age, as well as characteristics strictly connected with being teachers, such as previous training, experience in teaching, and perceived support. In summary, we found that, for both types of teachers, previous training in special education or ID, perceived support, and promotion of positive attitudes toward ID, in addition to the quality of the relationships with individuals with ID, are the factors that most affect the attitude toward ID. For special-education teachers only, the perceived efficacy of knowledge was also related to the attitude toward ID. On the contrary, previous experience teaching students plays a very limited role in affecting attitudes of mainstream teachers toward ID, and previous experience in teaching pupils with ID, even with severe/profound ID, was not found to be related to the attitude toward ID.

These findings highlight how the school and community context may promote the development of positive attitudes of teachers toward ID. Indeed, a school or community which can provide their teachers with high-quality training in disability, which is supportive in terms of social and material resources, and which allows the teachers to promote positive attitudes toward ID can foster favorable attitudes toward ID. Previous positive relationships of teachers with individuals with ID may reinforce these positive effects. A favorable disposition toward ID of the teachers may, in turn, stimulate the development of a positive attitude toward ID in their peers, as well as in other professionals working at the school. This would drastically increase the acceptance and the participation of students with ID in classrooms and social activities, in addition to their school and community inclusion, thereby improving their quality of life.

### Limitations and Future Directions

The participants of the present study were not selected randomly but based on their voluntary responses. Nevertheless, the participants were selected from 19 out of 20 Italian regions, represented 73 out of 107 Italian provinces, and were non statistically different from the study population in terms of gender, age, and school level taught. However, the method of selection based on voluntary response might have disproportionately selected teachers who were interested in the topic investigated. Indeed, most of the teachers who participated in this study reported having had positive prior contact with persons with ID, while none reported having had negative prior contact. This might have reduced the score variance in the ATTID questionnaire, thereby limiting the generalizability of the present results to teachers with a good predisposition toward ID. This indicates the need for further research of randomly selected individuals. However, this method of selection would require mandatory response to the questionnaire by all teachers; this is not feasible and would likely return answers that are not thoroughly sincere.

In accordance with previous studies in the narrow field of teachers’ attitudes toward the school inclusion of students with disabilities [31,34,56], the variance in attitude scores as explained by individual characteristics was quite low. Therefore, other variables might significantly affect teachers’ attitudes toward ID. Examples of possible variables are teachers’ assumptions or prejudice toward individuals with ID. Also, dimensions of teachers’ personality should be taken into account and, in particular, the openness to experience Big Five personality traits, which was found to have the greatest effect on social attitudes [57], such as those toward individuals with mental disorders [58] or autism spectrum disorder [59]. Identifying these factors is fundamental to implementing interventions aimed at changing negative attitudes toward ID.

## 5. Conclusions

Despite these limitations, the current study provides a detailed overview of attitudes toward ID, as well as of the related factors, for mainstream and special-education teachers who teach at different school levels in a country such as Italy, with a long history of school inclusion (approximately 50 years). This study shows how school and community contexts are relevant in promoting favorable attitudes toward ID, which are essential for the development of an inclusive environment for students with ID and, therefore, for the improvement of their quality of life.

## Figures and Tables

**Table 1:** Characteristics of the mainstream and special-education teachers.

	Mainstream Teachers (*n* = 307)	Special-Education Teachers (*n* = 237)
Age (%)		
18–29	4	4
30–39	14	32
40–49	35	42
50–59	38	17
60+	9	5
Quantity of contact with individuals with ID (Score range: 1–7)		
Mean (SD)	4.01 (1.69)	4.66 (1.58)
Range	1–7	1–7
Closeness of relationships with individuals with ID ^1^ (%)		
Neighbors, offspring’s schoolmates, individuals met in leisure activities or sports	3	1
Individuals met for volunteer or work activities	73	79
Relatives	12	11
Family members	12	9
Quality of relationships with individuals with ID ^1^ (%)		
Neutral	8	3
Good	70	60
Excellent	22	36
School level taught (%)		
Preschool	12	10
Elementary school	34	28
Middle school	23	30
High school	31	32
Training in special education or ID (Score range: 1–7)		
Mean (SD)	2.99 (1.81)	4.56 (1.82)
Range	1–7	1–7
Years of teaching experience (%)		
<5	11	28
5–10	11	27
10–20	38	32
20+	40	13
Years of experience teaching pupils with ID (%)		
<5	36	41
5–10	24	28
10–20	25	22
20+	15	8
Experience teaching pupils with severe/profound ID (%)		
Yes	60	72
No	40	28
Perceived efficacy of ID knowledge (Score range: 8–40)		
Mean (SD)	28.12 (5.64)	31.81 (4.30)
Range	8–40	8–40
Perceived support (Score range: 7–35)		
Mean (SD)	24.15 (4.33)	24.30 (4.58)
Range	8–35	10–35
Promotion of positive attitudes toward ID (Score range: 5–25)		
Mean (SD)	18.70 (2.42)	18.49 (2.43)
Range	11–25	10–25

^1^ The number of mainstream teachers and special-education teachers was 293 and 232, respectively, because 14 and 5 of them, respectively, reported not having had any experience with individuals with intellectual disability (ID).

**Table 2:** Means and standard deviations of raw score and 5-point Likert scale score obtained by mainstream teachers and special-education teachers for each dimension of the Attitudes Toward Intellectual Disability (ATTID) questionnaire.

	Mainstream Teachers				Special-Education Teachers			
	Raw Score		5-Point Likert Scale Score		Raw Score		5-Point Likert Scale Score	
ATTID Dimension	Mean	SD	Mean	SD	Mean	SD	Mean	SD
Discomfort	32.83	9.78	1.93	0.58	30.04	8.13	1.77	0.48
Knowledge of capacity and rights	45.61	9.23	2.28	0.46	43.77	9.63	2.19	0.48
Interaction	36.71	9.21	2.04	0.51	33.92	8.26	1.88	0.46
Sensitivity/Tenderness	10.02	3.85	2.50	0.96	9.47	3.56	2.37	0.89
Knowledge of causes of ID	16.49	4.11	2.36	0.59	16.11	3.67	2.30	0.52

**Table 3:** Correlation coefficients between general and teacher-specific characteristics and each dimension of the ATTID questionnaire for mainstream teachers and special-education teachers.

	ATTID Dimensions									
	Discomfort		Knowledge of Capacity and Rights		Interaction		Sensitivity/Tenderness		Knowledge of Causes of ID	
Individual’s Characteristics	Mainstream Teachers	Special-Education Teachers	Mainstream Teachers	Special-Education Teachers	Mainstream Teachers	Special-Education Teachers	Mainstream Teachers	Special-Education Teachers	Mainstream Teachers	Special-Education Teachers
General										
Age	0.03	0.02	0.26 **	0.16 *	0.10	0.14 *	−0.04	−0.05	0.07	0.05
Quantity of contact with individuals with ID	−0.43 **	−0.24 **	0.00	0.11	−0.27 **	−0.17 *	−0.29 **	−0.18 **	−0.04	−0.15 *
Closeness of relationships with individuals with ID ^1^	−0.10 °	0.08	0.04	−0.02	−0.11 *	−0.02	−0.07	0.09	−0.03	−0.16 *
Quality of relationships with individuals with ID ^1^	−0.47 **	−0.30 **	−0.08	−0.11	−0.38 **	−0.37 **	−0.33 **	−0.17 **	−0.02	−0.19 **
Teacher-specific										
School level taught	0.02	−0.04	−0.13 *	0.03	−0.03	0.09	0.04	−0.04	0.09	−0.05
Training in special education or ID	−0.23 **	−0.23 **	−0.04	0.08	−0.19 **	−0.12 °	−0.25 **	−0.25 **	−0.05	0.03
Years of teaching experience	0.06	−0.07	0.20 **	0.20 **	0.14 *	0.05	−0.07	−0.12 °°	0.09	−0.01
Years of experience teaching pupils with ID	−0.08	−0.10	0.08	0.23 **	−0.03	0.08	−0.16 **	−0.12 °°	−0.03	−0.05
Experience teaching pupils with severe/profound ID	−0.18 **	−0.09	0.02	0.15*	−0.10	−0.02	−0.14 *	−0.12 °°	0.01	0.04
Perceived efficacy of ID knowledge	−0.37 **	−0.38 **	−0.11 °°	−0.06	−0.31 **	−0.29 **	−0.27**	−0.17 **	−0.10	−0.02
Perceived support	−0.09	−0.14 *	−0.11 °	−0.18 **	−0.26 **	−0.25 **	0.02	0.05	−0.07	0.04
Promotion of positive attitudes toward ID	−0.07	−0.16 *	−0.11 *	−0.14 **	−0.13 *	−0.19 **	0.08	0.01	−0.24 **	−0.19 **

^1^ The number of mainstream teachers and special-education teachers was 293 and 232, respectively, because 14 and 5 of them, respectively, reported not having had any experience with individuals with ID. ** *p* ≤ 0.01; * *p* ≤ 0.05; °°° *p* ≤ 0.06; °° *p* ≤ 0.07; ° *p* ≤ 0.08.

**Table 4:** Hierarchical multiple regression analyses of general and teacher-specific characteristics for each dimension of the ATTID questionnaire for mainstream teachers and special-education teachers.

	ATTID Dimensions									
	Discomfort ^1^		Knowledge of Capacity and Rights		Interaction ^1^		Sensitivity/Tenderness ^1^		Knowledge of Causes of ID	
	Mainstream Teachers	Special-Education Teachers	Mainstream Teachers	Special-Education Teachers	Mainstream Teachers	Special-Education Teachers	Mainstream Teachers	Special-Education Teachers	Mainstream Teachers^2^	Special-Education Teachers^1^
**Individual’s Characteristics**	*β*	*β*	*β*	*β*	*β*	*β*	*β*	*β*	*β*	*β*
Step 1										
General										
Age	-	-	0.26 ***	0.16 *	-	0.21 **	-	-	-	-
Quantity of contact with individuals with ID	−0.28 ***	−0.17 *	-	-	−0.13 *	−0.10	−0.17 **	-0.13	-	−0.12
Closeness of relationships with individuals with ID	0.04	-	-	-	−0.01	-	-	-	-	−0.13 *
Quality of relationships with individuals with ID	−0.37 ***	−0.25 ***	-	-	−0.33 ***	−0.36 ***	−0.26 *	-0.12	-	−0.13
Adjusted *R^2^*	0.27 ***	0.10 ***	0.06 ***	0.02 *	0.15 ***	0.17 ***	0.13 ***	0.03 *	-	0.05 **
ANOVA *F*	36.83 ***	14.31 ***	21.29 ***	6.08 *	17.77 ***	16.25 ***	22.19 ***	4.69 *	-	5.25 **
Step 2										
General										
Age	-	-	0.23 **	0.08	-	0.23 **	-	-	-	-
Quantity of contact with individuals with ID	−0.24 ***	−0.06	-	-	−0.11	−0.03	−0.14 *	-0.07	-	−0.10
Closeness of relationships with individuals with ID	0.03	-	-	-	−0.05	-	-	-	-	−0.12 *
Quality of relationships with individuals with ID	−0.33 ***	−0.14 *	-	-	−0.25 ***	−0.28 ***	−0.24 ***	-0.09	-	−0.09
Teacher-specific										
School level taught	-	-	−0.14 *	-	-	-	-	-	-	-
Training in special education or ID	−0.04	−0.08	-	-	−0.03	-0.09	−0.15 *	−0.24 **	-	-
Years of teaching experience	-	-	0.02	−0.02	0.12 *	-	-	0.01	-	-
Years of experience teaching pupils with ID	-	-	-	0.14	-	-	−0.06	0.07	-	-
Experience teaching pupils with severe/profound ID	−0.01	-	-	0.09	-	-	0.01	-0.01	-	-
Perceived efficacy of ID knowledge	−0.10	−0.26***	−0.07	-	−0.09	−0.15 *	-0.01	-0.04	-	-
Perceived support	-	−0.09	−0.07	−0.13	−0.17 **	−0.19 **	-	-	-	-
Promotion of positive attitudes toward ID	-	−0.02	−0.11	−0.12	−0.06	−0.04	-	-	−0.24 ***	−0.16 *
Adjusted *R^2^*	0.27 ***	0.17 ***	0.09 ***	0.07* *	0.21 ***	0.22 ***	0.14 ***	0.05 **	0.0 5***	0.07 ***
ANOVA *F*	19.37 ***	8.68 ***	5.94 ***	3.92 **	10.46 ***	10.24 ***	8.82 ***	2.80 **	18.20 ***	5.55 ***
*sr^2^* incremental	0.01	0.08 ***	0.04 *	0.07 **	0.07 ***	0.07 **	0.03	0.04	-	0.02*

^1^ The number of mainstream teachers and special education teachers was 293 and 232, respectively, because 14 and 5 of them, respectively, reported not having had any experience with individuals with ID. ^2^ Simple linear regression. *** *p* ≤ 0.001; ** *p* ≤ 0.01; * *p* ≤ 0.05.

## Supplementary Materials

The following are available online at https://www.mdpi.com/1660-4601/17/19/7325/s1, Note S1: Description of the item modifications for the Italian adaptation of the Attitudes toward Intellectual Disability Questionnaire (ATTID); Table S1: Questions to investigate general characteristics; Table S2: Questions to investigate teachers-specific characteristics.

## References

[1] American Psychiatric Association (2013). Diagnostic and Statistical Manual of Mental Disorders.

[2] Schalock R.L., Borthwick-Duffy S.A., Bradley V.J., Buntinx W.H.E., Coulter D.L., Craig E.M., Gomez S.C., Lachapelle Y., Luckasson R., Reeve A. (2010). Intellectual Disability: Diagnosis, Classification, and Systems of Supports.

[3] World Health Organization ICD-11 for Mortality and Morbidity Statistics. https://icd.who.int/browse11/l-m/en#/.

[4] Schalock R.L., Verdugo M.A. (2002). Handbook on Quality of Life for Human Service Practitioners.

[5] Schalock R.L., Luckasson R., Tassé M.J. (2019). The contemporary view of intellectual and developmental disabilities: Implications for psychologists. Psicothema.

[6] Cagran B., Schmidt M. (2011). Attitudes of Slovene teachers towards the inclusion of pupils with different types of special needs in primary school. Educ. Stud..

[7] Ainscow M., César M. (2006). Inclusive education ten years after Salamanca: Setting the agenda. Eur. J. Psychol. Educ..

[8] European Agency for Special Needs and Inclusive Education (2014). Five Key Messages for Inclusive Education: Putting Theory into Practice.

[9] Srivastava M., De Boer A., Pijl S.J. (2013). Inclusive education in developing countries: A closer look at its implementation in the last 10 years. Educ. Rev..

[10] UNESCO (2009). Policy Guidelines on Inclusion in Education.

[11] Avramidis E., Kalyva E. (2007). The influence of teaching experience and professional development on Greek teachers’ attitudes towards inclusion. Eur. J. Spéc. Needs Educ..

[12] Dessemontet R.S., Bless G. (2013). The impact of including children with intellectual disability in general education classrooms on the academic achievement of their low-, average-, and high-achieving peers. J. Intellect. Dev. Disabil..

[13] UNESCO (1990). World Declaration on Education for All.

[14] UNESCO (1994). The Salamanca Statement and Framework for Action on Special Needs Education.

[15] (2001). No Child Left Behind. https://www.congress.gov/bill/107th-congress/house-bill/1/text.

[16] United Nations (2006). Convention on the Rights of Persons with Disabilities.

[17] Verdugo M.A., Navas P., Gómez L.E., Schalock R.L. (2012). The concept of quality of life and its role in enhancing human rights in the field of intellectual disability. J. Intellect. Disabil. Res..

[18] (1977). Law 517. https://www.gazzettaufficiale.it/eli/id/1977/08/18/077U0517/sg.

[19] (1992). Law 104. https://www.gazzettaufficiale.it/eli/id/1992/02/17/092G0108/sg.

[20] (2019). Law 96. https://www.gazzettaufficiale.it/eli/id/2019/08/28/19G00107/SG.

[21] Lauchlan F., Fadda R., Boyle C., Topping K.J. (2012). The “Italian model” of full inclusion: Origins and current directions. What Works in Inclusion?.

[22] Eagly A.H., Chaiken S. (1993). The Psychology of Attitudes.

[23] Rosenberg M.J., Hovland C.I., Rosenberg M., Hovland C., McGuire W., Abelson R., Brehm J. (1960). Cognitive, affective, and behavioral components of attitude. Attitude Organization and Change.

[24] Avramidis E., Norwich B. (2002). Teachers’ attitudes towards integration/inclusion: A review of the literature. Eur. J. Spéc. Needs Educ..

[25] De Boer A., Pijl S.J., Minnaert A. (2011). Regular primary schoolteachers’ attitudes towards inclusive education: A review of the literature. Int. J. Incl. Educ..

[26] Scruggs T.E., Mastropieri M.A. (1996). Teacher Perceptions of Mainstreaming/Inclusion, 1958–1995: A Research Synthesis. Except. Child..

[27] Hassanein E.E.A. (2014). Changing Teachers’ Negative Attitudes Toward Persons With Intellectual Disabilities. Behav. Modif..

[28] Özer D., Nalbant S., Aǧlamıș E., Baran F., Samut P.K., Aktop A., Hutzler Y. (2012). Physical education teachers’ attitudes towards children with intellectual disability: The impact of time in service, gender, and previous acquaintance. J. Intellect. Disabil. Res..

[29] Dessemontet R.S., Morin D., Crocker A.G. (2014). Exploring the Relations between In-service Training, Prior Contacts and Teachers’ Attitudes towards Persons with Intellectual Disability. Int. J. Disabil. Dev. Educ..

[30] Wilson C., Woolfson L.M., Durkin K. (2019). The impact of explicit and implicit teacher beliefs on reports of inclusive teaching practices in Scotland. Int. J. Incl. Educ..

[31] Ahmmed M., Sharma U., Deppeler J. (2012). Variables affecting teachers’ attitudes towards inclusive education in Bangladesh. J. Res. Spéc. Educ. Needs.

[32] Balboni G., Pedrabissi L. (2000). Attitudes of Italian teachers and parents toward school inclusion of students with mental retardation: The role of experience. Educ. Train. Ment. Retard. Dev. Disabil..

[33] Memisevic H., Hodzic S. (2011). Teachers’ attitudes towards inclusion of students with intellectual disability in Bosnia and Herzegovina. Int. J. Incl. Educ..

[34] Malinen O.-P., Savolainen H., Xu J. (2012). Beijing in-service teachers’ self-efficacy and attitudes towards inclusive education. Teach. Teach. Educ..

[35] Ojok P., Wormnæs S. (2013). Inclusion of pupils with intellectual disabilities: Primary school teachers’ attitudes and willingness in a rural area in Uganda. Int. J. Incl. Educ..

[36] Cornoldi C., Terreni A., Scruggs T.E., Mastropieri M.A. (1998). Teacher Attitudes in Italy After Twenty Years of Inclusion. Remedial Spéc. Educ..

[37] Morin D., Crocker A.G., Beaulieu-Bergeron R., Caron J. (2012). Validation of the attitudes toward intellectual disability - ATTID questionnaire. J. Intellect. Disabil. Res..

[38] International Test Commission (2017). International Test Commission Guidelines for Translating and Adapting Tests, 2nd ed. https://www.intestcom.org/files/guideline_test_adaptation_2ed.pdf.

[39] Schermelleh-Engel K., Moosbrugger H., Müller H. (2003). Evaluating the fit of structural equation models: Tests of significance and descriptive goodness-of-fit measures. Methods Psychol. Res..

[40] Jackson D.L., Gillaspy J.A., Purc-Stephenson R. (2009). Reporting practices in confirmatory factor analysis: An overview and some recommendations. Psychol. Methods.

[41] Evers A., Hagemeister C., Høstmælingen A., Lindley P., Muñiz J., Sjöberg A. (2013). EFPA Review Model for the Description and Evaluation of Psychological and Educational Tests. Test Review Form and Notes for Reviewers, Version 4.2.6. http://www.efpa.eu/download/650d0d4ecd407a51139ca44ee704fda4.

[42] Cicchetti D., Koenig K., Klin A., Volkmar F.R., Paul R., Sparrow S. (2010). From Bayes Through Marginal Utility to Effect Sizes: A Guide to Understanding the Clinical and Statistical Significance of the Results of Autism Research Findings. J. Autism Dev. Disord..

[43] Tabachnick B.G., Fidell L.S. (2013). Using Multivariate Statistics.

[44] Kaiser H.F., Rice J. (1974). Little Jiffy, Mark Iv. Educ. Psychol. Meas..

[45] Cortina J.M. (1993). What is coefficient alpha? An examination of theory and applications. J. Appl. Psychol..

[46] Bobbio A., Manganelli A.M. (2011). Measuring social desirability responding. A short version of Paulhus’ BIDR 6. Test. Psychom. Methodol. Appl. Psychol..

[47] Paulhus D.L., Robinson J.P., Shaver P.R., Wrightsman L.S. (1991). Measurement and Control of Response Bias. Measures of Personality and Social Psychological Attitudes.

[48] European Commission Eurostat (2020). Classroom Teachers and Academic Staff by Education Level, Programme Orientation, Sex and Age Groups.

[49] Cohen J. (1988). Statistical Power Analysis for the Behavioral Sciences.

[50] King B.M., Minium E.W. (2003). Statistical Reasoning in Psychology and Education.

[51] Benjamini Y., Hochberg Y. (1995). Controlling the False Discovery Rate: A Practical and Powerful Approach to Multiple Testing. J. R. Stat. Soc. Ser. B Stat. Methodol..

[52] Faul F., Erdfelder E., Lang A.-G., Buchner A. (2007). G*Power 3: A flexible statistical power analysis program for the social, behavioral, and biomedical sciences. Behav. Res. Methods.

[53] Faul F., Erdfelder E., Buchner A., Lang A.-G. (2009). Statistical power analyses using G*Power 3.1: Tests for correlation and regression analyses. Behav. Res. Methods.

[54] Huberty C.J., Petoskey M.D., Tinsley H.E.A., Brown S.D. (2000). Multivariate Analysis of Variance and Covariance. Handbook of Applied Multivariate Statistics and Mathematical Modeling.

[55] Olson C.L. (1979). Practical considerations in choosing a MANOVA test statistic: A rejoinder to Stevens. Psychol. Bull..

[56] Savolainen H., Engelbrecht P., Nel M., Malinen O.-P. (2011). Understanding teachers’ attitudes and self-efficacy in inclusive education: Implications for pre-service and in-service teacher education. Eur. J. Spéc. Needs Educ..

[57] McCrae R.R. (1996). Social consequences of experiential openness. Psychol. Bull..

[58] Wolska A., Malina A. (2020). Personality and attitudes towards people with mental disorders: Preliminary studies results. Int. J. Soc. Psychiatry.

[59] Gillespie-Lynch K., Daou N., Sanchez-Ruiz M.-J., Kapp S.K., Obeid R., Brooks P.J., Someki F., Silton N., Abi-Habib R. (2019). Factors underlying cross-cultural differences in stigma toward autism among college students in Lebanon and the United States. Autism.

