# Supplementary material for: Attitudes of Mainstream and Special-Education Teachers toward Intellectual Disability in Italy: The Relevance of Being Teachers
Source: Int J Environ Res Public Health. 2020 Oct 7;17(19):7325. doi: 10.3390/ijerph17197325 (PMC7579515; doi:10.3390/ijerph17197325)
Supplement: Supplementary file 1 [file ijerph-17-07325-s001.pdf]

Supplementary Materials

Note S1

Description of the Item Modifications for the Italian Adaptation of the Attitudes toward Intellectual Disability Questionnaire (ATTID)

The original item “In your opinion, the majority of individuals with intellectual disabilities should have the opportunity of attending a regular secondary school” was split into the two following items: “In your opinion, the majority of individuals with intellectual disabilities should have the opportunity of attending a regular middle school” and “In your opinion, the majority of individuals with intellectual disabilities should have the opportunity of attending a regular high school.” The two following items were added: “In your opinion, the majority of individuals with intellectual disabilities should have the opportunity of attending a regular preschool” and “In your opinion, the majority of individuals with intellectual disabilities should have the opportunity of attending university”.

Table S1

Questions to Investigate General Characteristics

1. Age
   - ☐ 18-29 years old
   - ☐ 30-39 years old
   - ☐ 40-49 years old
   - ☐ 50-59 years old
   - ☐ 60 years or older

2. Indicate how many individuals with ID you know or have met *(write the number)*:

   ____

3. DURING YOUR LIFETIME, you have had contacts or interactions with individuals with ID:
   - ☐ Very often
   - ☐ Often
   - ☐ Sometimes
   - ☐ Never

4. Individuals with ID that you know are (*you can mark more than one answer)*:
   - ☐ Family members
   - ☐ Relatives
   - ☐ Neighbors
   - ☐ People for whom you do volunteer work
   - ☐ People for whom you work (students, clients, users)
   - ☐ Your child’s schoolmates
   - ☐ People you have met during leisure or sporting activities
   - ☐ Other (please, specify):____

5. You would describe the relationships with individuals with ID who you know:

☐ Excellent

☐ Good

☐ Neutral

☐ Bad

☐ Very bad

Table S2

Questions to Investigate Teachers-Specific Characteristics

1. Currently, you work in a:

   ☐ Preschool

   ☐ Elementary school

   ☐ Middle school

   ☐ High school

2. You have a specialization for special education activities:

   ☐ Yes

   ☐ No

3. If yes, please specify the type of specialization:

   ____

4. Indicate the approximately overall number of hours spent attending training courses in ID or IDD:

   ☐ None

   ☐ 1-10 hours

   ☐ 10-29 hours

   ☐ 30-50 hours

   ☐ > 50 hours

5. Indicate the years of your teaching experience:

   ☐ < 5 years

   ☐ 5-10 years

   ☐ 10-20 years

   ☐ > 20 years

6. Indicate the years of experience teaching students with ID:

- ☐ < 5 years
- ☐ 5-10 years
- ☐ 10-20 years
- ☐ > 20 years

7. You have experience teaching students with severe/profound ID:

- ☐ Yes
- ☐ No

	Totally agree	Agree	Neither agree or disagree	Disagree	Totally disagree
8. Do you think that your knowledge about ID is sufficient for:					
a. identifying the characteristics of pupils with ID	☐	☐	☐	☐	☐
b. identifying educational needs of pupils with ID	☐	☐	☐	☐	☐
c. adapting educational goals for pupils with ID	☐	☐	☐	☐	☐
d. adapting educational material for pupils with ID	☐	☐	☐	☐	☐
e. encouraging learning in pupils with ID	☐	☐	☐	☐	☐
f. fostering relationships of pupils with ID with their peers with typical development	☐	☐	☐	☐	☐
g. handling the behavior of pupils with ID	☐	☐	☐	☐	☐
9. You feel able to promote an effective process of teaching /learning for pupils with ID	☐	☐	☐	☐	☐

10. You believe:

a. that the activities carried out by educators, psychologists and other professionals represent a help for your profession	☐	☐	☐	☐	☐
b. that the services and local resources represent a help for your profession	☐	☐	☐	☐	☐
c. having the necessary resources (e.g. human, financial, temporal) and materials (e.g. books, videos, technology devices) to teach pupils with ID	☐	☐	☐	☐	☐
d. being sufficiently supported in teaching to pupils with ID by your school's administrative clerks	☐	☐	☐	☐	☐
e. being sufficiently supported in teaching to pupils with ID by your school's teacher colleagues	☐	☐	☐	☐	☐
f. being sufficiently supported in teaching to pupils with ID by your school's professionals (e.g. educators, psychologists)	☐	☐	☐	☐	☐
g. that your school promotes the school inclusion of pupils with ID	☐	☐	☐	☐	☐
h. that the attitude toward ID affects school and social inclusion of individuals with ID	☐	☐	☐	☐	☐

	i. that reading narrative fictions and watching movies about ID may modify attitudes toward individuals with ID	☐	☐	☐	☐	☐
11.	In your opinion, to promote an effective teaching/learning process to pupils with ID is necessary:					
	a. having specific characteristics and predispositions	☐	☐	☐	☐	☐
	b. having attended numerous training courses	☐	☐	☐	☐	☐
	c. being trained on a specific method	☐	☐	☐	☐	☐
